# Annual acknowledgement of BMC medicine manuscript reviewers

**DOI:** 10.1186/s12916-015-0271-1

**Published:** 2015-02-09

**Authors:** Sabina Alam

**Affiliations:** BioMed Central Ltd, 236 Gray’s Inn Road, London, WC1X 8HB UK

## Abstract

The editors of *BMC Medicine* would like to extend our sincere thanks to all our reviewers who have contributed to the journal in Volume 12 (2014). The time and expertise they have provided is greatly appreciated.

**Ali Abbasi**

Netherlands

**Rebecca Abbott**

Australia

**Frances Aboud**

Canada

**Bo Abrahamsen**

Denmark

**Harold Adams**

USA

**Alastair Ager**

USA

**Devendra Agrawal**

USA

**Golo Ahlenstiel**

Australia

**Nasser Al-Daghri**

Saudi Arabia

**Waleed Alhazzani**

Canada

**Mohammad Ali**

USA

**Kristina Allen-Brady**

USA

**Rieke Alten**

Germany

**Doug Altman**

UK

**Gloria Alvarez-Llamas**

Spain

**Juan-Manuel Anaya**

Colombia

**Hans-Joachim Anders**

Germany

**Ranjit Mohan Anjana**

India

**Nicholas Anstey**

Australia

**Judith Arnetz**

USA

**Armando Arredondo**

Mexico

**Richard Ashcroft**

UK

**Dagfinn Aune**

UK

**Kari Auranen**

Finland

**Philippe Autier**

France

**Rasmi Avula**

India

**Helen Ayles**

Zambia

**Laurent Azoulay**

Canada

**Kathryn Backholer**

Australia

**Sunil Badve**

USA

**William Baker**

USA

**Marian Bakermans-Kranenburg**

Netherlands

**Padmapriya Banada**

USA

**Hector Barajas Martinez**

USA

**Alyssa Barry**

Australia

**Elizabeth Barry**

USA

**Giovanni Battista Bartolucci**

Italy

**Diego Bassani**

Canada

**John Batsis**

USA

**Michael Bauer**

Germany

**Jeannette Beasley**

USA

**Clemens Becker**

Germany

**James Beeson**

Australia

**Philip Bejon**

UK

**Jimmy Bell**

UK

**Ezequiel Bellorin-Font**

Venezuela

**Derrick Bennett**

UK

**Thierry Berghmans**

Belgium

**James Bernat**

USA

**Sonja Berndt**

USA

**Franco Berrino**

Italy

**Zulfiqar Bhutta**

Pakistan

**Roland Bingisser**

Switzerland

**Giuseppe Biondi-Zoccai**

Italy

**Michael Blaha**

USA

**Miri Blank**

Israel

**Zachary Bloomgarden**

USA

**Sally Blower**

USA

**Richard Bohannon**

USA

**Dale Bond**

USA

**Stefanos Bonovas**

Greece

**Amy Borenstein**

USA

**Claudia Borreani**

Italy

**Cristina Bosetti**

Italy

**Luigi Bouchard**

Canada

**Pierre Bougneres**

France

**Matthew Boulton**

USA

**Paul Bourbeau**

USA

**Nash Boutros**

USA

**Peter Bower**

UK

**Cynthia Boyd**

USA

**Bernard Brabin**

UK

**Su Brailsford**

UK

**Jonathan Braun**

USA

**Lorraine Brennan**

Ireland

**Emilio Bria**

Italy

**Gordon Broderick**

Canada

**Martin Brüne**

Germany

**Hermine Brunner**

USA

**Jonathan Burns**

South Africa

**Howard Burris**

USA

**Barbara Burtness**

USA

**Peter Byass**

Sweden

**Christopher Callahan**

USA

**David Cameron**

UK

**Cristina Campoy**

Spain

**Giovanni Cancarini**

Italy

**Gideon Caplan**

Australia

**Chris Carlsten**

Canada

**Virginia Carrieri-Kohlman**

USA

**Giulio Cavalli**

Italy

**Sandra Cesario**

USA

**Steven Chadban**

Australia

**Christine Chambers**

Canada

**David Champion**

Barbados

**Jeremy Chapman**

Australia

**Abhijit Chaudhuri**

UK

**Tanya Chikritzhs**

Australia

**Davide Chiumello**

Italy

**Gerardo Chowell**

USA

**Robert Christensen**

USA

**George Chrousos**

Greece

**Andrea Cipriani**

UK

**Duncan Clark**

USA

**Michael Clark**

UK

**Peter Clifton**

Australia

**Jane Coad**

UK

**Marco Colleoni**

Italy

**Simon Collin**

UK

**Helena Cortez-Pinto**

Portugal

**Carol Coupland**

UK

**Pim Cuijpers**

Netherlands

**Timothy Dall**

USA

**Gennaro D'Amico**

Italy

**Matija Daniel**

UK

**Kaberi Dasgupta**

Canada

**Nicholas Day**

Thailand

**Peter de Jonge**

Netherlands

**Michel de Lorgeril**

France

**Sandrine de Ribaupierre**

Canada

**Luc de Witte**

Netherlands

**Lisa DeAngelis**

USA

**Charles Decarli**

USA

**Agnes Dechartres**

France

**Suha Deen**

UK

**Jean-Marie Degryse**

Belgium

**Vittorio Demicheli**

Italy

**Youping Deng**

USA

**Daniel Denis**

USA

**Marc Denis**

France

**Patrick Dessein**

South Africa

**Augusto Di Castelnuovo**

Italy

**Massimo Di Maio**

Italy

**Eleftherios Diamandis**

Canada

**Paul Dickman**

Sweden

**Diederik Dippel**

Netherlands

**Jodie Dodd**

Australia

**Denise Doolan**

Australia

**Therese Dowswell**

UK

**Pat Doyle**

UK

**Tommaso Dragani**

Italy

**Mark Dransfield**

USA

**Joost Drenth**

USA

**Stephen Duffy**

UK

**Venkata Duvvuri**

Canada

**Johanna Dwyer**

USA

**Walter Dzik**

USA

**Kenneth Eames**

UK

**Julian Eaton**

Togo

**Solvig Ekblad**

Sweden

**Vikki Entwistle**

UK

**Susan Everson-Rose**

USA

**Pam Factor-Litvak**

USA

**David Faeh**

Switzerland

**Karl Fagerstrom**

Sweden

**Rita Faria**

UK

**Deborah Fein**

USA

**Eva Fenwick**

Australia

**Alfio Ferlito**

Italy

**Marcelo Ferreira**

Brazil

**Emilio Ferrer**

USA

**Eduardo Ferriolli**

Brazil

**Edith Feskens**

Netherlands

**Jess Fiedorowicz**

USA

**Guido Filler**

Canada

**Milton Finegold**

USA

**Susan Fiscus**

USA

**Abraham Flaxman**

USA

**Martin Fleisher**

USA

**Mary Fletcher**

USA

**Cor Jesus Fontes**

Brazil

**Betsy Foxman**

USA

**Giovanny Franca**

Brazil

**Massimo Franchini**

Italy

**Kenneth Freedland**

USA

**Michael Frenneaux**

UK

**Matthew Friedman**

USA

**Jan Friedrich**

Canada

**Susanne Fuessel**

Germany

**Harold Furr**

USA

**Takahisa Furuta**

Japan

**Annemarie Gagnon**

Canada

**Gennaro Galasso**

Italy

**Catharine Gale**

UK

**Daozhou Gao**

USA

**Michel Garenne**

France

**Amit Garg**

Canada

**Paul Garner**

UK

**Gerald Gartlehner**

Austria

**Colin Geddes**

UK

**Chiara Gentili**

Italy

**Blaise Genton**

Switzerland

**Steven George**

USA

**Davina Ghersi**

Australia

**Panteleimon Giannakopoulos**

Switzerland

**Peter Giannoudis**

UK

**Ruth Gilbert**

UK

**Frank Gilliland**

USA

**Ian Gilmore**

UK

**James Giordano**

USA

**Gary Giovino**

USA

**John Gladman**

UK

**Thomas Glass**

USA

**Stephen Glasser**

USA

**Lise Gluud**

Denmark

**Danijela Gnjidic**

Australia

**Michael Gollob**

Canada

**Takahisa Gono**

Japan

**Allan Gottschalk**

USA

**Pierre Goussard**

South Africa

**William B Grant**

USA

**Darren C Greenwood**

UK

**Marie Griffin**

USA

**Wolfgang Grisold**

Austria

**Sandeep Grover**

India

**Danan Gu**

USA

**Bruno Gualano**

Brazil

**Giovanni Guaraldi**

Italy

**Leonor Guariguata**

Belgium

**Iva Gunnarsson**

Sweden

**Lars L Gustafsson**

Sweden

**Erik Gylfe**

Sweden

**Balazs Györffy**

Hungary

**Mary Haan**

USA

**Walter Haas**

Germany

**Peter Hajek**

UK

**Andrew Hall**

UK

**Elizabeth Halloran**

USA

**Shannon Halloway**

USA

**John Halperin**

USA

**Julien Haroche**

France

**Frank Harrell**

USA

**Nicholas Hart**

UK

**Elaine Hay**

UK

**Joel Hay**

USA

**Colleen Hayes**

USA

**Kirstie Haywood**

UK

**Hiddo Heerspink**

Netherlands

**Philippe Hernigou**

France

**Philip Hill**

New Zealand

**Anke Hinney**

Germany

**Timothy Hofer**

USA

**Michael Hoffmeister**

Germany

**Bruce Hollis**

USA

**Geoffrey Holmes**

UK

**Thomas Allan House**

UK

**Gang Hu**

USA

**Frank Hu**

USA

**Holly Hull**

USA

**Susan Hutfless**

USA

**Brian Hutton**

Canada

**Jane Hutton**

UK

**Ahmed Idbaih**

France

**Rob Imrie**

UK

**Anne Jackson**

UK

**Graham Jackson**

UK

**K.S. Jacob**

India

**Edward Janus**

Australia

**Bach Jean-Francois**

France

**Prakash Mohan Jeena**

South Africa

**Pavla Jendelova**

Czech Republic

**David Jenkins**

Canada

**Prabhat Jha**

Canada

**Joaquin Jimenez**

USA

**Markus Jokela**

Finland

**Hayley Jones**

UK

**Jessica Jones-Smith**

USA

**Somchai Jongwutiwes**

Thailand

**Susan Jordan**

Australia

**Matthew Jose**

Australia

**Jonathan Juliano**

USA

**Siripen Kalayanarooj**

Thailand

**Bonnie Kaplan**

Canada

**Aaron Katz**

USA

**Mark Katz**

USA

**Justin Keen**

UK

**Cyril Kendall**

Canada

**Andre Pascal Kengne**

Cameroon

**Ronald Kessler**

USA

**Hector Keun**

UK

**Carl Kirkwood**

Australia

**George Kirov**

UK

**Niranjan Kissoon**

Canada

**Jos Kleijnen**

UK

**Dhanpat Kochar**

India

**Clemens Kocken**

Netherlands

**Paul Komenda**

Canada

**Suminori Kono**

Japan

**Amos Korczyn**

Israel

**Boris Kotchoubey**

Germany

**Shyam Kottilil**

USA

**Anthony Kovac**

USA

**Reinhard Krausz**

Canada

**Alexander Kulminski**

USA

**Anton Kunst**

Netherlands

**Andreas Kurtz**

Germany

**Anne Stine Kvehaugen**

Norway

**Dirk Lachenmeier**

Germany

**Andre Lalonde**

Canada

**Scott Langevin**

USA

**Peter Langhorne**

UK

**Simon Langley-Evans**

UK

**Catharina Lavebratt**

Sweden

**Carl Lavie**

USA

**Michael Lawrence**

USA

**David Le Couteur**

Australia

**Jinseok Lee**

South Korea

**Peter Lee**

UK

**Hongxing Lei**

China

**Michael Leitzmann**

USA

**Trudo Lemmens**

Canada

**Grigorios Leontiadis**

Canada

**Andres G. Lescano**

Peru

**Rona Levy**

USA

**Joel Lexchin**

Canada

**Chin-Shang Li**

USA

**Wenbin Liang**

Australia

**Judith Lichtman**

USA

**Eric Lien**

USA

**David J Lim**

USA

**Jakob Linseisen**

Germany

**Francesca Little**

South Africa

**Andrew Liu**

USA

**Xiaoan Liu**

China

**Fiona Lobban**

UK

**Yoon Kong Loke**

UK

**Alan Lopez**

Australia

**Jenny Guek-Hong Low**

Singapore

**Robert Luben**

UK

**Andreas Lundh**

Denmark

**Knut E A Lundin**

Norway

**Adrian Luty**

France

**Kristine Lykens**

USA

**Ethel Maciel**

Brazil

**Nick Macklon**

UK

**Markus Maeurer**

Sweden

**Kathryn Maitland**

UK

**Mark Manary**

USA

**Samuel Mann**

USA

**Claus Manniche**

Denmark

**Martí Manyalich**

Spain

**William Manzanares**

Uruguay

**Vincent Marchesi**

USA

**Fabrizio Marcucci**

Italy

**Vitaly Margulis**

USA

**Staffan Marild**

Sweden

**Jose Marin**

Spain

**Maurie Markman**

USA

**Hugh Markus**

UK

**Francesco Marongiu**

Italy

**Thomas Marrie**

Canada

**Miguel Martinez-Gonzalez**

Spain

**Luca Mascitelli**

Italy

**Joseph Massaro**

USA

**Colin Mathers**

Switzerland

**Paolo Matricardi**

Italy

**Richard Maude**

Thailand

**Peter Maxwell**

UK

**Harriet Mayanja**

Uganda

**Oleg Mayboroda**

Netherlands

**James McCarthy**

Australia

**Sarah McDonald**

Canada

**Leah McGrath**

USA

**Chris McManus**

UK

**David McManus**

USA

**Lisa McShane**

USA

**David Menkes**

New Zealand

**Usha Menon**

UK

**Stewart Mercer**

UK

**Stefano Merler**

Italy

**Margaret Miller**

Australia

**Virginia M. Miller**

USA

**Edward Mills**

Canada

**Martin Miner**

USA

**Alec Miners**

UK

**Roberto Miniati**

Italy

**Kazutoyo Miura**

USA

**Eiji Miyoshi**

Japan

**David Moher**

Canada

**Ben Willem Mol**

Netherlands

**Ann Moormann**

USA

**Nils Morgenthaler**

Germany

**Ralph Mösges**

Germany

**Alan Moss**

USA

**Alfredo Mota**

Portugal

**Miranda Mugford**

UK

**Zindoga Mukandavire**

UK

**Matthew Muldoon**

USA

**Majon Muller**

Netherlands

**Javier Munoz**

USA

**Michael Murphy**

UK

**Robert L. Murphy**

USA

**Dedee Murrell**

Australia

**Theonest Mutabingwa**

Tanzania

**Stella Muthuri**

UK

**John Myburgh**

Australia

**Janet Myers**

USA

**Luis Nacul**

UK

**VasI Naganathan**

Australia

**John Nemunaitis**

USA

**Marie-Louise Newell**

UK

**Marie Ng**

USA

**Gregory Nichols**

USA

**Ellen Niederberger**

Germany

**Dorte Lisbet Nielsen**

Denmark

**Tuomo Nieminen**

Finland

**George Ntaios**

Greece

**Kimberly O'Brien**

USA

**Nobuaki Ochi**

Japan

**Daniel O'Connor**

Australia

**Stephen O'Leary**

Australia

**Adrienne O'Neil**

Australia

**Bregje D Onwuteaka-Philipsen**

Netherlands

**Peter O'Sullivan**

Australia

**Jose A Oteo**

Spain

**Jan Erik Otterstad**

Norway

**Karen Ousey**

UK

**Betty Pace**

USA

**Balakrishnan Pachamuthu**

India

**Tibor Palfai**

USA

**Panagiotis Papanagiotou**

Germany

**Carmine Pariante**

UK

**Jana Parizkova**

Czech Republic

**Anna Peeters**

Australia

**Margaret Pepe**

USA

**Kenneth Perkins**

USA

**Ivan Perry**

Ireland

**Lars Åke Persson**

Sweden

**Vanamail Perumal**

India

**Jaime Peters**

UK

**Mark Peters**

UK

**William Petri**

USA

**John Petrie**

UK

**Daniel Petrovic**

Slovenia

**Olivia Phung**

USA

**Antonio Picarelli**

Italy

**Michael Pichichero**

USA

**Marco Alessandro Pierotti**

Italy

**Bertram Pitt**

USA

**Lucilla Poston**

UK

**Hollis Potter**

USA

**John Powles**

UK

**Alexandra Prados-Torres**

Spain

**Martin Preisig**

Switzerland

**Theodore Price**

USA

**Giancarlo Pruneri**

Italy

**Milo Puhan**

USA

**Chengxuan Qiu**

Sweden

**Petros Rafailidis**

Greece

**James Raftery**

UK

**Anita Raj**

USA

**Swapnil Rajpathak**

USA

**Taina Rantanen**

Finland

**Julie Ratcliffe**

Australia

**Ken Redekop**

Netherlands

**Jürgen Rehm**

Canada

**Eduardo Reis**

Brazil

**Jochen Reiser**

USA

**Jan Remme**

France

**Yves Renaudineau**

France

**Andrew Rhodes**

UK

**Fernando Rico-Villademoros**

Spain

**Kristin Riekert**

USA

**Christine Ritchie**

USA

**David Roberts**

UK

**Mark Robson**

USA

**Nicolas Roche**

France

**Mauricio Rodrigues**

Brazil

**Fernando Rodríguez-Artalejo**

Spain

**Sabine Rohrmann**

Switzerland

**Pedro Romero-Aroca**

Spain

**Pol Maria Rommens**

Germany

**Matthew Rosengart**

USA

**Christian Roth**

Germany

**Laurence Rubenstein**

USA

**Gerta Rücker**

Germany

**Elaine Rush**

New Zealand

**Lisa Rydén**

Sweden

**Farid Saad**

Germany

**Caroline Sabin**

UK

**Marcus Saemann**

Austria

**Monika Safford**

USA

**Richard Saitz**

USA

**Oliver Sakowitz**

Germany

**Emilia Salvadori**

Italy

**Elizabeth Sampson**

UK

**Helle Samuelsen**

Denmark

**David Sanders**

UK

**Hidefumi Sasaki**

Japan

**Shizuka Sasazuki**

Japan

**Sivasankaran Sashidharan**

UK

**Leslie Satin**

USA

**Roberta Scherer**

USA

**Jan Schieveld**

Netherlands

**Michael Schlander**

Germany

**Helen Schneider**

South Africa

**Peter Schofield**

Australia

**Marcus J Schultz**

Netherlands

**Kevin Schwartzman**

Canada

**Ralf Schwarzer**

Germany

**Guido Schwarzer**

Germany

**Andreas Scorilas**

Greece

**Kate Scott**

New Zealand

**Lori Scott-Sheldon**

USA

**Éric Senneville**

France

**Franco Servadei**

Italy

**Ruth Shalev**

Israel

**Dennis Shanks**

Australia

**Kenji Shibuya**

Japan

**Yehuda Shoenfeld**

Israel

**Yardena Siegman-Igra**

Israel

**Eleanor Singer**

USA

**Dhirendra Narain Sinha**

India

**Michael Skinner**

USA

**Tod Sloan**

USA

**Arjen Slooter**

Netherlands

**Robert Smith**

Canada

**Lynne Smith**

USA

**Yiqing Song**

USA

**Yong Song**

China

**Albert Sotto**

France

**Harold Sox**

USA

**Nicholas Spencer**

UK

**Alessio Squassina**

Italy

**Amanda Staiano**

USA

**Stephen Stansfeld**

UK

**David Steel**

UK

**Norbert Stefan**

Germany

**Guenther Steger**

Austria

**Steven Steinhubl**

USA

**Tim Stockwell**

Canada

**Jørund Straand**

Norway

**Tor Strand**

Norway

**Carolyn Summerbell**

UK

**Artur Swiergiel**

Poland

**A. Marcell Szasz**

Hungary

**Abd Tahrani**

UK

**Ambrose Talisuna**

Kenya

**Rajiv Tandon**

USA

**Yi-Wei Tang**

USA

**David Tanne**

Israel

**Linda Tapsell**

Australia

**Basil Tarlatzis**

Greece

**Anne Taylor**

Australia

**Bruce Taylor**

Australia

**Sophie Testa**

Italy

**Shakila Thangaratinam**

UK

**Roger Thomas**

Canada

**Paul Thompson**

USA

**Stephen Tong**

Australia

**Jorg Tost**

France

**Howard Trachtman**

USA

**Andrea Tricco**

Canada

**Shoichiro Tsugane**

Japan

**Mark Tuszynski**

USA

**Aage Tverdal**

Norway

**Reidar Tyssen**

Norway

**Dirk Ubbink**

Netherlands

**Esther Udina**

Spain

**Jaime Uribarri**

USA

**Dhananjay Vaidya**

USA

**Eleftherios Vamvakas**

USA

**Rob van Dam**

Singapore

**Marianne A.B. van der Sande**

Netherlands

**Mireille van Poppel**

Netherlands

**Isabel Varela-Nieto**

Spain

**Sander Veldhuyzen van Zanten**

Canada

**Sten Vermund**

USA

**Alessandro Vespignani**

USA

**Cecile Viboud**

USA

**Andrew Vickers**

USA

**Reinhold Vieth**

Canada

**Ian Viney**

UK

**Rüdiger von Kries**

Germany

**Jeremy Walker**

UK

**Neff Walker**

USA

**Sasiwarang Wannamethee**

UK

**Stuart Watson**

UK

**Helen Weatherly**

UK

**Connie Weaver**

USA

**W Douglas Weaver**

USA

**Thomas Wellems**

USA

**Paul Welsh**

UK

**Lisa White**

Thailand

**Nicholas John White**

Thailand

**Will Whiteley**

UK

**Susan Whiting**

Canada

**Heather Whitson**

USA

**Erik Wiemer**

Netherlands

**Thomas Williams**

Kenya

**Ira Wilson**

USA

**Philip Wilson**

UK

**Elizabeth Winzeler**

USA

**Federica Wolf**

Italy

**Evelyn Wong**

Australia

**Geoff Wong**

UK

**Vincent Wong**

Hong Kong

**Nicole Wright**

USA

**Lin Xu**

Hong Kong

**Hushan Yang**

USA

**Patsy Yates**

Australia

**Bu Yeap**

Australia

**Neville Yeomans**

Australia

**Kimberly Yonkers**

USA

**Bryan Young**

Canada

**Sera Young**

USA

**Raul Zamora-Ros**

Spain

**Marco Zappa**

Italy

**Maximilian Zeyda**

Austria

**Guangxiang Zhang**

USA

**Qi Zhou**

Canada

**Brian Zikmund-Fisher**

USA

**Robert Zivadinov**

USA

